# Role of standard echocardiography in Anderson–Fabry disease

**DOI:** 10.3389/fcvm.2024.1467815

**Published:** 2024-10-24

**Authors:** Maddalena Conte, Giuseppe Cioffi, Maria Grazia Romeo, Laura Petraglia, Erika Parente, Paolo Poggio, Veronika Myasoedova, Vincenzo Russo, Raffaella Lauro, Roberta Accardo, Dario Leosco, Valentina Parisi

**Affiliations:** ^1^ Department of Translational Medical Sciences, University of Naples Federico II, Naples, Italy; ^2^ Unit for the Study of Aortic, Valvular and Coronary Pathologies, Centro Cardiologico Monzino IRCCS, Milan, Italy; ^3^ Department of Biomedical, Surgery and Dental Sciences, University of Milan, Milan, Italy; ^4^ Cardiology and Syncope Unit, Department of Translational Medical Sciences, University of Campania “Luigi Vanvitelli”-Monaldi Hospital, Naples, Italy

**Keywords:** Anderson–Fabry disease, echocardiography, cardiomyopathy, risk stratification, cardiac imaging

## Abstract

Cardiac involvement strongly impacts prognosis in patients with Anderson–Fabry disease (AFD). All cardiac structures, such as the left ventricle and the left atrium, the aorta, the right sections, and the heart valves can be affected by morphological and functional abnormalities. Standard echocardiography has a crucial role in the characterization of AFD cardiomyopathy. Being a diffuse, non-invasive, easily reproducible, and inexpensive investigation, echocardiography represents the most appropriate tool for screening AFD cardiomyopathy. Furthermore, echocardiographic evaluation is the essential imaging method to support the physician also in the follow-up and risk stratification of AFD patients. Therefore, echocardiography is useful in all stages of the disease, both to reveal the first signs of cardiac involvement and to guarantee timely treatment in the preclinical stage and to estimate the extent of cardiac involvement, define possible complications, and evaluate the response to treatment in patients with established cardiomyopathy. The latest advanced echocardiographic techniques, such as speckle-tracking analysis, are offering new insights into the early detection of AFD cardiac involvement, thus suggesting a promising role for echocardiography in selecting appropriate candidates for treatment. In this review, we will examine the role of standard echocardiography in AFD, focusing on its use in screening for cardiac involvement, detailed characterization of AFD cardiomyopathy, and risk stratification of AFD patients.

## Introduction

Anderson–Fabry disease (AFD) is a rare X-linked lysosomal storage disorder caused by a deficiency of the *α*-galactosidase A lysosomal enzyme (*α*-Gal A), resulting in pathological accumulation of lysosomal globotriaosylceramide (Gb3) and related globotriaosylsphingosine (lyso-Gb3) in several tissues, leading to multiorgan involvement and high morbidity and mortality (1).

Two major clinical phenotypes are described: the type 1 classic phenotype and the milder, type 2 later-onset phenotype. Type 1 is prevalent in male patients and typically occurs in childhood or adolescence with early symptoms including acroparesthesias, angiokeratomas, hypohidrosis, and a characteristic corneal dystrophy. Type 2 later-onset AFD typically occurs later and often primarily involves the heart.

Type 1 phenotype is associated with a higher risk of multiorgan failure and premature mortality (2, 3).

Cardiac involvement strongly impacts on prognosis (4). Echocardiography is the first-line imaging tool in AFD, with an essential role in screening, clinical management, and prognostic stratification (5). Echocardiography has many advantages as it is widely diffused and reproducible, fast, non-invasive, and low-cost. Thus, AFD patients routinely undergo echocardiographic examinations, and evaluation of the images over time allows physicians to early detect abnormal structural changes and timely treat disease progression and possible complications.

**Figure 1:**
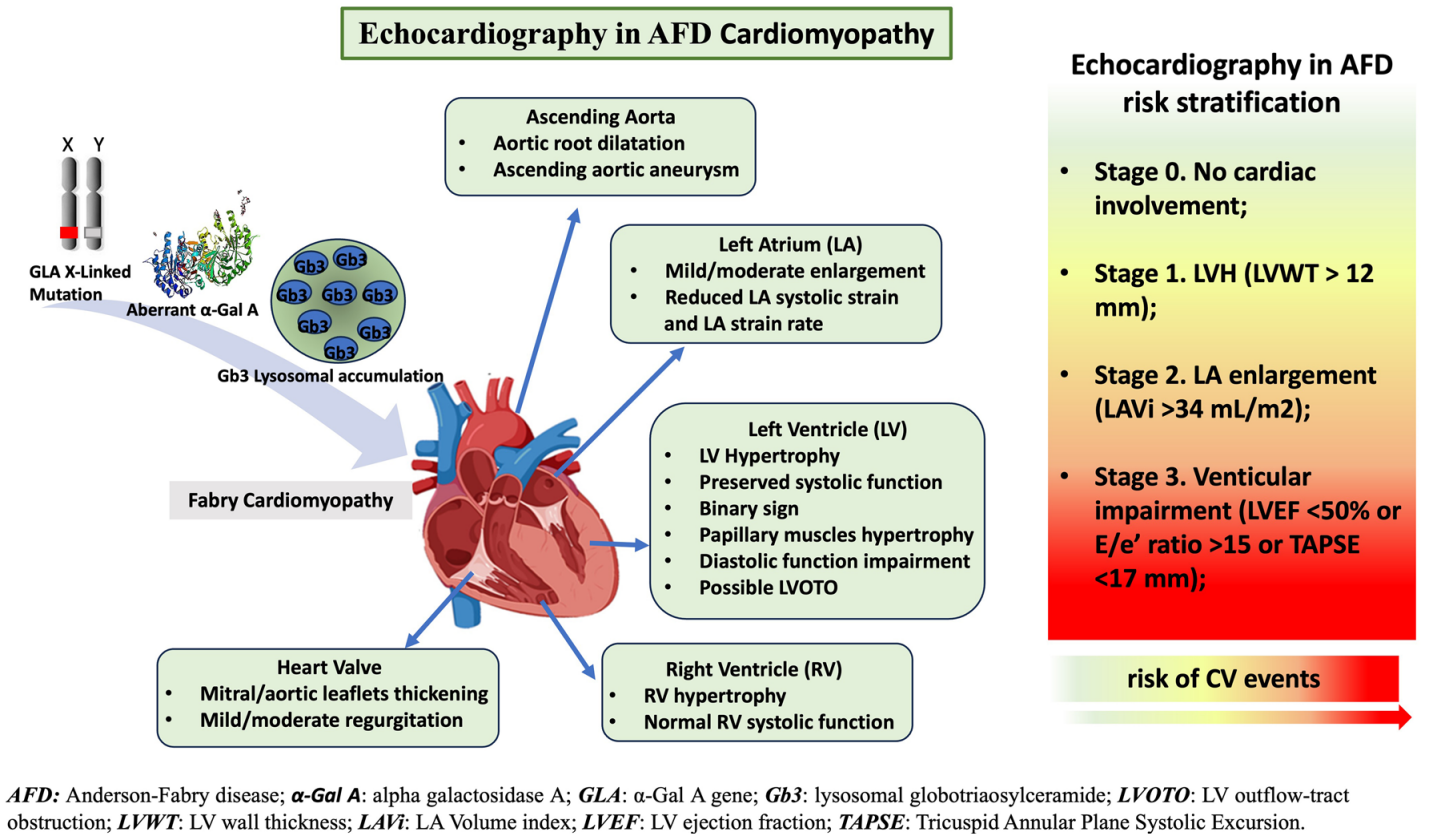
The role of standard-echocardiography in characterization of Anderson-Fabry disease (AFD)-cardiomyopathy and risk stratification. Standard echocardiography evaluation and its crucial role in AFD patients: echocardiography supports physician in the diagnosis, follow up and risk stratification of AFD patients.

In this review, we will deeply analyze the role of standard echocardiography in AFD, focusing on screening for cardiac involvement, AFD cardiomyopathy evaluation, and AFD risk stratification (Figure 1).

## Echocardiography in AFD screening

AFD cardiac involvement is the result of the accumulation of lysosomal globotriaosylceramide (Gb3) and related globotriaosylsphingosine (lyso-Gb3) in the heart, which in turn activate a chronic inflammatory process and autoimmunity (6–8). Following the introduction of enzyme replacement therapy (ERT), early recognition of AFD has become crucial to limit disease progression (9).

**Figure 2:**
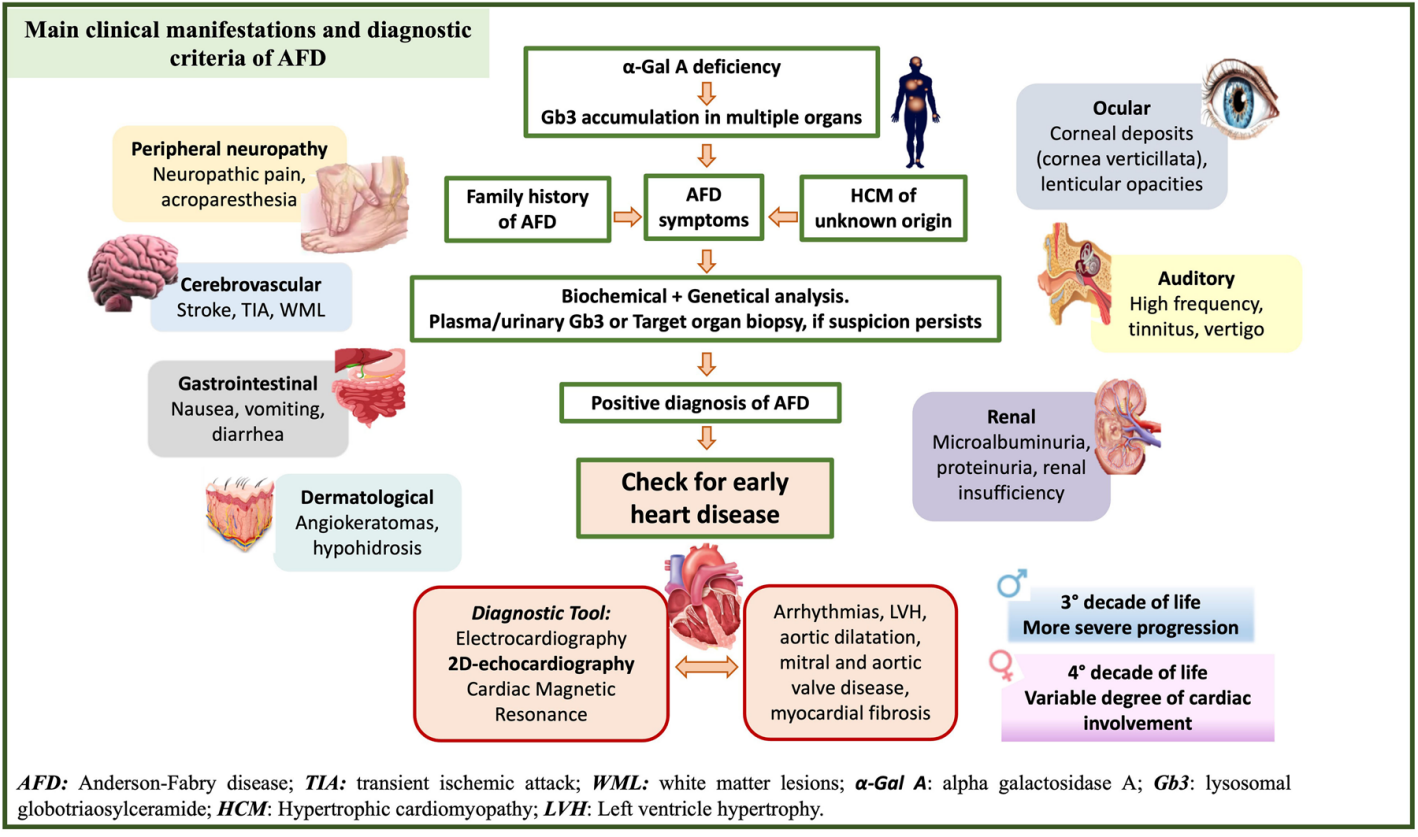
Clinical and diagnostic criteria of Anderson Fabry (AFD) disease and early detection of AFD-cardiomyopathy. Clinical manifestation, main signs and symptoms and diagnostic tools to support physicians in the diagnosis of AFD patients.

In these terms, echocardiography has a key role in the detection of early abnormalities. Recent guidelines from the European Society of Cardiology state that AFD should be suspected in patients with left ventricular hypertrophy (LVH) and additional cardiac and extracardiac red flags (10) (Figure 2). However, LVH is unlikely to be seen in patients aged <20 years, where the diagnosis is usually based on family history or other extracardiac symptoms.

In carriers of pathogenic variants, tissue Doppler imaging (TDI) and speckle tracking allow early detection of cardiac involvement independently of LVH (11). TDI strain and strain rate were reduced in AFD patients compared with normal control subjects (12). Zamorano et al. (13) showed, on both male and female patients with AFD, that TDI velocities were inversely related to left ventricular (LV) mass and demonstrated that abnormal TDI velocities were evident prior to the development of LVH in serial echocardiographic evaluations.

More recently, the speckle-tracking strain has been shown to identify AFD independently of LVH with greater sensitivity and specificity than TDI (14). The reduction of global longitudinal strain (GLS) is usually due to a regional decrease in longitudinal strain in the basal inferolateral region (15, 16). Of interest, the regional strain impairment has been shown to correlate in the same myocardial regions with late gadolinium enhancement (LGE) at cardiac magnetic resonance (CMR). Circumferential gradient strain reduction and a loss of the normal base-to-apex circumferential gradient were evident in both AFD patients with and without LVH (16).

These pieces of evidence support the role of echocardiographic TDI and speckle tracking in the early detection of AFD cardiac involvement and suggest a promising role for echocardiography in selecting appropriate candidates for treatment.

## Echocardiography in AFD cardiomyopathy

**Figure 3:**
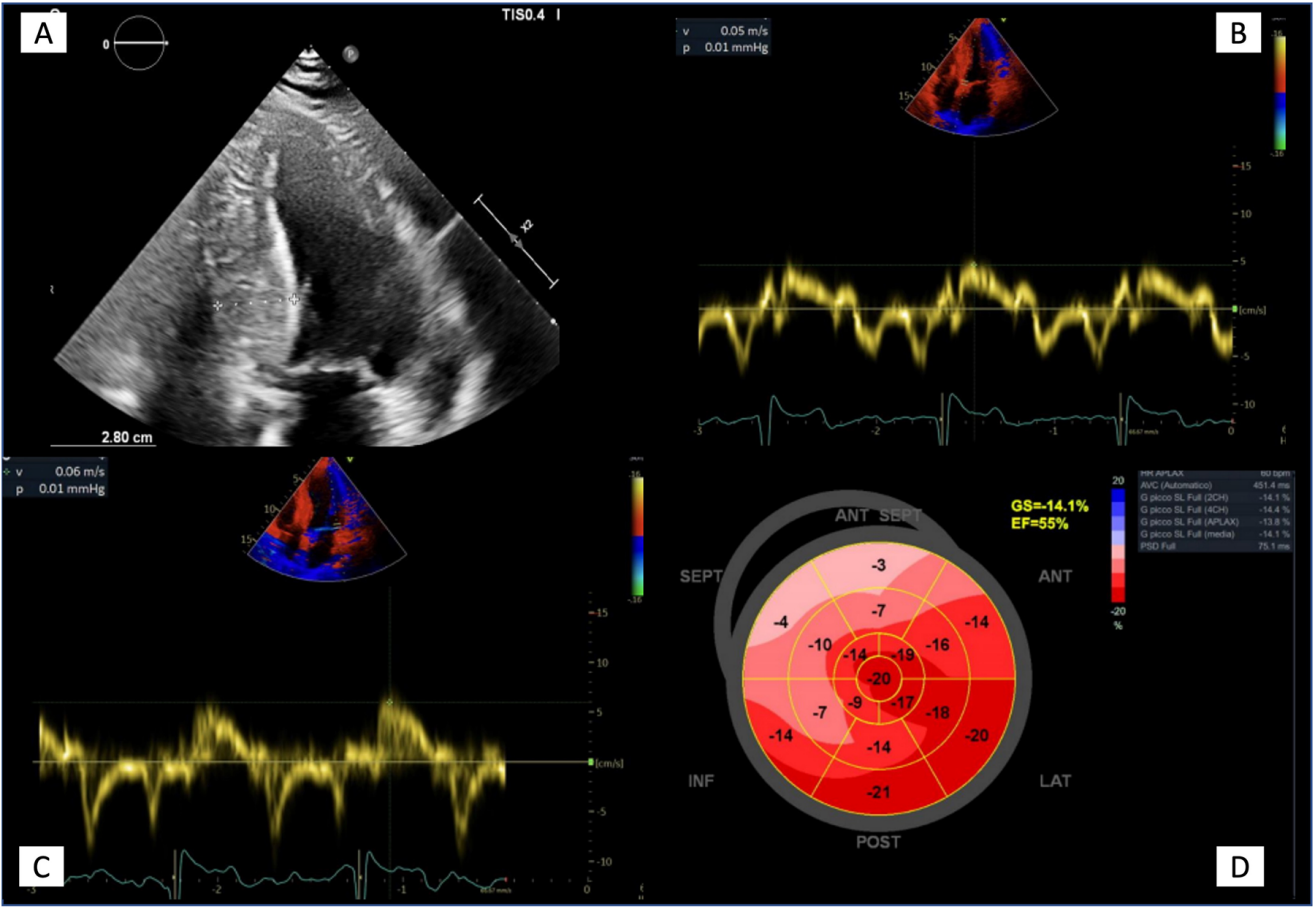
Echocardiographic features in advanced Anderson-Fabry disease cardiomyopathy. **(A)** Apical four chambers view showing the maximal wall thickness (28 mm) and the typical binary sign; **(B, C)** Tissue Doppler mitral annular velocities at septal and lateral corners respectively, showing low systolic velocities (5 and 6 cm/s respectively); **(D)** 2D speckle tracking analysis bull's eye plot, showing reduced LV GLS value (−14.1%).

Echocardiography is an essential tool in AFD patients and provides a detailed characterization of AFD cardiomyopathy. Cardiac alterations in AFD include structural and functional abnormalities of both the left and right ventricles (RV), left atrium (LA), aorta, and heart valves (Table 1; Figure 3).

**Table 1:** Echocardiographic features of AFD cardiomyopathy according to disease stage.

Structure	Early stages	Overt cardiomyopathy
Left ventricle	No LVH Preserved systolic function No Regional wall motion abnormalities Mild diastolic dysfunction Impaired TDI velocities and GLS	Increased wall thickness: concentric (most common), asymmetric septal, eccentric, apical Binary sign Papillary muscle thickening and hyperechogenicity Preserved systolic function until late stages Diastolic function often impaired Abnormal TDI findings (often before increase in wall thickness) Reduced GLS (often in the basal posterior/lateral segments) LVOTO or midventricular obstruction (not uncommon)
Left atrium	Abnormal TDI findings LA strain impaired	Mild to moderate atrial enlargement Increased atrial reversal velocities Abnormal TDI findings Reduced LA systolic strain and strain rate
Valves	No significant structural and functional abnormalities	Leaflet thickening and redundancy (especially in mitral and aortic valves) Valvular regurgitation (often mild, rarely significant)
Aorta	Arterial remodeling (increased intima–media thickness)	Aortic dilatation at the sinus of Valsalva and ascending aorta
Right ventricle	No RVH Normal TDI findings Normal TAPSE value Normal RV-GLS and RV-FWS	RVH Generally preserved systolic function, but severe dysfunction can occur Abnormal TDI findings RV-GLS and RV-FWS impaired despite normal systolic function

AFD, Anderson–Fabry disease; LVH, left ventricular hypertrophy; TDI, tissue Doppler imaging; GLS, global longitudinal strain; LV, left ventricle; LVOTO, left ventricular outflow tract obstruction; LA, left atrium; RVH, right ventricular hypertrophy; RV, right ventricle; TAPSE, tricuspid annular plane systolic excursion; RV-FWS, strain of RV free wall.

### Left ventricle

#### Hypertrophy

The echocardiographic study of the LV allows the evaluation of LV mass. An increased LV mass index (defined as >95 g/m^2^ for women and >115 g/m^2^ for men) identifies LVH (17). AFD represents an under-recognized cause of LVH, and an integrated approach is required for differential diagnosis, including clinical evaluation and a multi-imaging approach (Table 2).

**Table 2:** Clinical findings and multi-imaging approach for differential diagnosis in patients with LVH.

Disease	Clinical presentation	Echocardiography	ECG	CMR LGE	Histology
Fabry disease	- Young age at presentation (male: 11 ± 7 years; female: 23 ± 16 years) - Neuropathic pain - Impaired sweating - Skin rashes	- Symmetrical increase in LV and RV wall thickness (most common) - Preserved EF	- Increased or normal QRS complex voltage - Abnormal PR interval	- Focal - Midwall - Inferolateral wall	- Enlarged myocytes - Glycolipid deposits in lysosomes
Hypertrophic cardiomyopathy	- Young age at presentation - (17–18 years) - Asymptomatic - Angina - Dyspnea - Syncope - Sudden death	- Asymmetrical hypertrophy - Small LV cavity - LVOT obstruction - Preserved EF	- Increased QRS complex voltage - Pseudo-delta wave - Giant T-wave inversion	- Patchy - Midwall - Junctions of the ventricular septum and RV	- Myocyte hypertrophy - Myofibrillar alteration - Fibrosis
Hypertensive heart disease	- Adults - History of hypertension	- Symmetrical hypertrophy - Mild LV dilation - Preserved EF	- Increased QRS complex voltage - Non-specific ST-T-wave changes	- No pattern - Mainly subendocardial	- Enlarged myocytes - Increased myocyte nuclei concentration`

LVH, left ventricular hypertrophy; LV, left ventricle; RV, right ventricle; EF, ejection fraction; LVOTO, left ventricular outflow tract obstruction.

According to the current guidelines, AFD disease should be suspected in patients with LVH and additional cardiac and extracardiac red flags (10). LVH, commonly concentric (18), can be asymmetric with maximal wall thickness at the septal or apical level. However, at the early stages of the disease, LVH may be limited to the posterolateral basal wall (19, 20).

Differential diagnosis of LVH etiology is sometimes challenging, especially in the presence of other comorbidities that increase LV afterload. In these cases, a multi-imaging approach, including CMR, along with a careful clinical evaluation is required to guide the diagnosis and management.

CMR is often necessary for accurate volumetric and functional analysis and detailed myocardium characterization. CMR has a high spatial resolution, ideal for evaluating LV wall thickening patterns. Deva et al. (21) studied the spectrum of morphological phenotypes and CMR myocardial scar patterns in AFD. They demonstrated that concentric thickening and inferolateral mid-myocardial scarring are the most common manifestations of AFD; however, they reported a percentage of cases mimicking the morphological manifestations of hypertrophic cardiomyopathy, with apical and asymmetric septal hypertrophy. These phenotypes have more apical and midventricular LV scarring. CMR is also useful in the early diagnosis of AFD. Recent studies have identified non-contrast T1 mapping as an early marker of cardiac involvement in AFD, with high sensitivity and specificity (22–24).

#### LV systolic function

In the early stages of cardiac involvement, no impairment of systolic function is observed, and the LV ejection fraction (LVEF) is usually preserved or even supranormal (25). In the advanced stages of the disease, a reduction in LVEF can be observed, which is associated with a worse prognosis (26). Generally, there are no regional wall motion abnormalities, especially in the early stages of the disease. However, hypokinesia or akinesia of the LV posterior and inferior walls is described in some patients, as an expression of myocardial fibrosis.

#### Binary sign

Pieroni et al. (27) identified a specific echocardiographic feature of AFD called “binary sign” matching echocardiographic and histologic findings. They defined the binary sign as “the appearance of a clear black and white interface of the LV myocardium due to the adjacency of a bright, hyperechogenic region to a relatively low echo intensity region.” At histology, this finding was associated with endomyocardial glycosphingolipid compartmentalization, reflecting a peculiar feature of AFD. However, other authors raised doubts about the clinical utility of the binary sign as this feature can be non-specific. Kounas et al. (28) reported the binary sign in 21% of hypertrophic cardiomyopathy (HCM) patients without AFD. Also, Koskenvuo et al. (29) reported a lack of sensitivity and specificity of binary signs in AFD patients. Overall, the heterogenicity of AFD phenotypic variation makes applicability of binary sign of limited utility, but it can have a role in the differential diagnosis of unexplained LVH.

#### Papillary muscle hypertrophy

Echocardiographic findings of papillary muscle (PM) hypertrophy and trabecular complexity support the diagnosis of AFD cardiac involvement (30). However, PM hypertrophy is not specific to AFD and is present also in Friedreich ataxia, amyloidosis, and hypertensive patients (31). In the parasternal short axis, both PMs can be seen simultaneously. Through the measurement of both PM areas (manually traced from a short-axis view at the papillary muscle level) and LV cavity circumference using the endocardial border, it is possible to calculate the PM/LV ratio. AFD patients present a significantly higher PM/LV ratio than hypertensive, amyloidosis, and Friedreich patients (31). In hypertensive patients, the high wall stress leads also to a progressive LV cavity dilation, so relative PM/LV does not increase over time. Also in Friedreich ataxia, the end-diastolic wall thickness is not more than 14 mm, and PM size does not increase as much (32). Thus, in AFD cardiomyopathy, LV and papillary hypertrophy in combination with a small LV cavity lead to an increased absolute and relative PM/LV ratio which results in a useful, although non-specific marker.

#### LV outflow tract obstruction (LVOTO)

In contrast to hypertrophic cardiomyopathy, LVOTO has a very low incidence in patients with AFD (33). In AFD patients, LVOTO appears to be the result of a small LV cavity and PM hypertrophy and can be diagnosed during exercise echocardiography. Calcagnino et al. (34) studied exercise-induced LVOTO in AFD symptomatic patients. LVOTO was measured using continuous-wave Doppler in the apical five-chamber view with a latent obstruction defined as a peak LV outflow tract (LVOT) gradient >50 mmHg during or after exercise. This was the first report of a provocable LVOTO in symptomatic AFD patients. Graziani et al. (35) reported a case series of patients with advanced cardiac involvement at diagnosis who developed LVOTO during follow-up, despite optimal therapy. They described that LVOTO was mainly due to the extension and distribution of the LVH, rather than asymmetric septal basal hypertrophy and mitral valve abnormalities.

### Left atrium and diastolic function

Fibrosis and increased LV wall thickness, typical of AFD cardiomyopathy, are associated with progressive impairment of diastolic function (36), although restrictive pathophysiology is observed rarely and only in the most advanced stages of the disease. In AFD, the accumulation of glycolipids occurs intracellularly, and not in the interstitium, as happens in infiltrative disorders, such as amyloidosis. This different pathophysiology would explain why restrictive physiology is a frequent feature in infiltrative disorders but uncommon in AFD (37).

Assessment of myocardial relaxation with TDI function is a reliable method for early identification of preclinical AFD cardiomyopathy, even before the development of LVH (38). AFD patients, regardless of LVH, have a significant reduction of systolic (S’), early diastolic (e’), and late diastolic (a’) velocities, compared with normal control subjects. In AFD patients, the Ea/Aa ratio is reduced, and the E/e’ ratio is higher compared to control subjects (39).

Diastolic dysfunction, related to myocardial fibrosis, is frequent in patients with AFD. Septal E/e’ ratio has been described as the best echocardiographic marker suggestive of LGE at CMR. At the same time, LGE is detected even in the absence of measurable cardiac functional impairments, suggesting that diastolic dysfunction would not be a prerequisite for LGE in AFD (40).

Histological evaluation of the atrial myocardium demonstrated glycolipid accumulation, which appears related to LA dilation, dysfunction, and arrhythmias (41).

Recent studies have highlighted an increase in LA size in AFD patients, even before the development of LVH, and offered new insights into LA functional impairment in AFD patients. In a study by Boyd et al., LA volume was increased in AFD patients without LVH and normal diastolic function. This evidence suggests that the early stages of the pathological process may be associated with alterations in atrial myocardial properties and that measurements of LA size and function may be useful in the early diagnosis of AFD, before the development of LVH (42). Therefore, atrial myopathy may be independent of diastolic dysfunction and LVH.

In a retrospective cohort study, LA strain, strain rates, and phasic LA volumes were studied in 50 patients with AFD and compared with 50 healthy control subjects. In AFD, the LA reservoir, conduit, and contractile functions by speckle-tracking echocardiography were all found to be affected (43).

Although there are no robust data on the role of LA strain, some authors (44) highlighted the usefulness of left atriopathy in discriminating AFD and amyloidosis, both cardiomyopathies with a hypertrophic phenotype. These authors demonstrated that transthyretin cardiac amyloidosis is characterized by more advanced LA structural and functional remodeling compared to patients with AFD and similar degree of LVH, suggesting that left atriopathy is less important in AFD than in amyloidosis when comparing patients with the same degree of LVH.

### Valvular heart disease

It is increasingly recognized that cardiomyopathies and valvular heart diseases may share different pathophysiological mechanisms. Several genetic or acquired diseases, such as storage or immune-mediated disorders, can affect both the myocardium and the valves, with important prognostic and therapeutic implications (45).

The glycolipid deposition typical of AFD has also been found at the level of the heart valves, and valvular infiltration leads to leaflets thickening and deformation (46).

Although all the cardiac valves can be affected, Linhart et al. (18) reported that structural abnormalities most frequently involve the mitral and aortic valves. The mitral valve is the most affected, especially in young patients which usually has mild regurgitation and leaflets thickening. After the age of 40 years, alterations also occur in the aortic valve. Aortic valve involvement and the presence of valve abnormalities are associated with a more advanced stage of AFD.

Mild aortic and mitral regurgitation is frequently observed, especially in advanced stages of AFD. Lillo et al. (47) raised the hypothesis of deposition of glycolipids also in the subvalvular apparatus and reported a case of isolated chordal rupture without valve leaflet prolapse. Evidence of aortic stenosis is a very rare finding in AFD. Vlachou et al. (48) analyzed at histology of the aortic valve of an AFD patient, reporting a loss of valve architecture with edema, myxoid degeneration, and calcification. Giustino et al. (49) first reported a case of low gradient aortic valve stenosis in an elderly patient with AFD later undergoing transcatheter aortic valve implantation (TAVI).

Nowadays, few cases of patients with AFD treated surgically for severe aortic stenosis have been reported in the literature; nonetheless, all agree on the clinical benefit of treatment even during enzyme therapy. Overall, valve involvement in AFD appears to be frequent but rarely requires surgical intervention (48, 49).

### Aortic dilation

Aortic root dilation is listed among the clinical features of AFD (50). The excessive accumulation of glycolipids in the aortic media and in the small arterioles causes degenerative changes in the vessel wall. The hypertrophy of the media and the marked vacuolization of the smooth muscle cells also determine the involvement of the aortic root in patients with AFD, as demonstrated by biochemical studies carried out postmortem (51).

Echocardiographic dilation of the aortic root is often found in patients with AFD (52). Ascending aortic dilatation appears to be independent of other cardiovascular risk factors (53); however, in patients with dilatation of aortic root, the ascending aorta dilation is associated with LVH, suggesting a relationship between an advanced stage of AFD and vascular remodeling.

Barbey et al. (54) assessed that ascending aortic dilation occurred frequently in AFD male patients compared with the normal population. Female patients with AFD developed dilation of the sinus of Valsalva and ascending aorta less frequently than males, and approximately 15–20 years later. These authors did not observe dilation at the descending aorta in any patient.

The most serious complications of aneurysms of the ascending aorta are dissection and rupture; although no cases of aortic emergencies have been reported in AFD patients, the presence of aortic dilation must be closely monitored to prevent such events (36).

### Right sections

Several studies investigated the extent of RV involvement in AFD patients and reported that the RV is frequently and progressively affected, with a prevalence ranging between 31% and 71% (55, 56).

Niemann et al. performed standard echocardiography in 75 genetically confirmed consecutive AFD patients and described RV hypertrophy in 53 patients (71%). The authors found a significant positive correlation between LV and RV wall thickness. Interestingly, the degree of RV involvement correlated with the stage of LV cardiomyopathy (56).

In a study conducted by Kampmann et al. (55), 28% of patients with RV hypertrophy had RV dysfunction, expressed by tricuspid annulus movement <10 mm and a prolonged RV pre-ejection period/pulmonary ejection time ratio or pseudo-normal or restrictive RV filling patterns. Interestingly, in these patients, the severity of RV dysfunction correlated with the extent of LVH.

In line with this evidence, Palecek et al. (57) found RV hypertrophy in 40% of AFD patients, with similar prevalence in both genders. They identified the right ventricular hypertrophy (RVH) feature with preserved systolic but impaired diastolic function as the typical RV structural change in AFD. Indeed, they described RV systolic dysfunction in only 4.3% of FD patients with RVH, while diastolic dysfunction was highlighted in 47% of examined patients.

RVH therefore does not appear to significantly influence RV systolic function, although a slight reduction in RV TDI systolic velocity values can be observed in patients with RVH (58). Systolic velocities at TDI might be helpful in the differential diagnosis of infiltrative heart disease: AFD patients have better RV systolic function compared with those with cardiac amyloidosis with similar levels of RV thickness (58).

Interestingly, the tricuspid annular plane systolic excursion (TAPSE), a parameter for the evaluation of global RV function, was found to be normal even in the advanced stages of AFD cardiomyopathy, making it not very useful for the evaluation of RV involvement in this population (56).

Although conflicting evidence, RV speckle-tracking echocardiography and RV strain may highlight initial RV involvement in AFD patients, even when conventional ultrasound parameters are within normal ranges, with worst RV systolic function parameters in overt cardiomyopathy (59–61).

So far, there is limited evidence regarding right atrial dysfunction in AFD patients. However, it has been reported that right atrial strain values are slightly decreased in AFD patients. Mattig et al. (62) found that alterations in the strain parameters of the right heart, both atrial and ventricular, were more pronounced in patients with cardiac amyloidosis compared to those with AFD, as already highlighted for the LV. Thus, the integration of structural and functional parameters of the right and left heart could be useful to discriminate AFD and amyloidosis.

To date, there are no consistent data on the clinical implications of RV involvement in AFD. A recent study (63) evaluated a possible correlation between RV hypertrophy and dysfunction and major clinical events, showing a significant association between RV hypertrophy and systolic function with clinical outcomes in AFD. However, the authors highlighted that only proteinuria and LVH emerged as independent predictors of outcome in AFD patients, thus suggesting that RV involvement may represent a useful marker of advanced disease but does not influence prognosis in AFD patients.

## Gender differences in Anderson–Fabry disease

Gender differences in AFD are primarily due to the nature of X-linked inheritance. Males, who are hemizygous for the mutated gene, typically experience more severe disease manifestations than females, who are heterozygous and may exhibit a wider range of clinical phenotypes. Sex differences in cardiac manifestations are described. Heterozygote female carriers have been reported to be affected by both cardiac and extracardiac manifestations, and it therefore appears that AFD should be considered an X-linked dominant disease (64). Males experience an earlier onset and more severe progression of cardiomyopathy, such as LVH, diastolic dysfunction, and valvular abnormalities. Because of X-chromosome inactivation, female carriers can have variable disease expression, with later symptoms and a variable degree of cardiac involvement, often without significant LVH and only mild echocardiographic changes, with less GLS involvement (3, 18). Regarding LVH, in females, the severity of LVH is strongly correlated with increasing age (65).

A cross-sectional study by Niemann et al. (66) enrolled a large AFD cohort, including 58 female and 46 male patients. In this population, LVH, regional myocardial deformation, and myocardial fibrosis were assessed by standard echocardiography, strain rate imaging, and LGE-CMR. No significant differences have been reported in the ejection fraction and diastolic parameters between female and male patients and respective sex-matched controls. The LV wall thickness was significantly higher in AFD male patients. While only 17% of female patients showed LVH, 65% of male patients had LVH. LGE was detected in 48% of male patients, interestingly all with LV wall thickness >12 mm. In contrast, in females, LGE was detected in 33% of women, consistently in the posterolateral basal wall and in the absence of increased LV wall thickness, suggesting that female patients may develop fibrosis without showing LVH. Therefore, assessment of replacement fibrosis should be included in the clinical management of AFD patients (66).

Gender differences have also been described in vascular remodeling and aortic dilation, which is experienced less frequently in women and typically about 15–20 years later (54).

## Role of standard echocardiography in risk stratification

LVH is a major phenotypic expression of cardiac involvement in AFD and has been considered a risk marker for cardiovascular events and heart failure (67, 68). LVH is considered the strongest prognostic marker in AFD cardiomyopathy and the main cardiac marker to monitor the treatment efficacy (69, 70)_._ More recently, a comprehensive echocardiographic evaluation has been used to classify AFD cardiac involvement into four stages associated with cardiovascular outcomes. In 314 patients with genetically confirmed AFD, Meucci et al. (5) proposed the following staging: Stage 0, patients without cardiac involvement; Stage 1, LVH, defined as a maximal LV wall thickness >12 mm; Stage 2, LA enlargement, defined as LA volume index >34 mL/m^2^; and Stage 3, LV systolic or diastolic dysfunction, defined as LV ejection fraction <50% or diastolic dysfunction measured by E/e’ ratio ≥15 or TAPSE <17 mm. The study endpoint was the composite of all-cause death, hospitalization for heart failure, new-onset atrial fibrillation, major bradyarrhythmias or tachyarrhythmias, and ischemic stroke. Interestingly, although worsening stages of cardiac damage were accompanied by greater LVH, the association with cardiovascular events was more robust with the use of the proposed stages than maximal LV wall thickness alone. These findings suggest that a comprehensive echocardiography evaluation has a significant role in AFD patient risk stratification.

Sometimes it is useful to integrate the diagnostic process with CMR. Morphological and functional study of the heart with CMR and GLE seems to be useful in identifying AFD patients who are at high risk of adverse cardiac events, regardless of established clinical risk factors. Thus, CMR seems to be an important tool for the risk stratification of this population (67). CMR is also important for therapeutic strategies, as the presence of GLE is a major biomarker of AFD disease and is one of the indicators that allows the financed enzyme replacement therapy (71).

## Role of standard echocardiography in monitoring the effects of treatment

Intravenous enzyme replacement therapy (ERT) with agalsidase-alfa, agalsidase-beta, or oral chaperone therapy (migalastat) represents the specific treatments for AFD, to reduce symptoms and improve survival (72).

However, the benefit of treatment seems to be significant only in the early stages of the disease. Therapy started later may only slow the progression of disease, without affecting already existing organ damage, such as cardiomyopathy, which may become irreversible. Advanced AFD cardiomyopathy, defined by the coexistence of LVH, myocardial fibrosis, and severely reduced regional LV function, indicates a poor response to therapy. Thus, echocardiography represents a main tool, together with other indicators, for monitoring the response to AFD-specific treatment (73).

One of the most used parameters to monitor the effects of ERT is the change in LV mass (74). A reduction in LV mass and an improvement in LV function has been described after 1 year of ERT in a small cohort of AFD patients, thus highlighting a short-term effect of the treatment (12). The long-term effects of ERT on AFD cardiomyopathy appear to be related to the extent of myocardial fibrosis at the time of treatment initiation. In patients without detectable fibrosis and mild hypertrophy at baseline, normalization of LV wall thickness and mass during ERT was observed, as well as improvement to normalization of LV radial and longitudinal septal function. These patients experienced benefits also in terms of exercise capacity.

Strain rate imaging seems to be superior to global parameters such as ejection fraction in monitoring ERT treatment. An increase in radial strain rate after 1 year of ERT appears to predict long-term improvement in regional myocardial function. In contrast, a reduction in lateral longitudinal function is an unfavorable sign in the advanced stages of the disease and predicts an adverse outcome (73).

## Conclusions

Standard echocardiography evaluation has a crucial role in AFD patients. Echocardiography is a readily available and diffuse imaging methodology that supports physicians in the diagnosis, follow-up, and risk stratification of AFD patients.
